# The 2D Hotelling filter - a quantitative noise-reducing principal-component filter for dynamic PET data, with applications in patient dose reduction

**DOI:** 10.1186/1756-6649-13-1

**Published:** 2013-04-10

**Authors:** Jan Axelsson, Jens Sörensen

**Affiliations:** 1 Department of Radiation Sciences, Radiation Physics, Umeå University, Umeå, Sweden; 2 PET-center, Department of Radiology, Oncology and Radiation Sciences, Uppsala University, Uppsala, Sweden

## Abstract

**Background:**

In this paper we apply the principal-component analysis filter (Hotelling filter) to reduce noise from dynamic positron-emission tomography (PET) patient data, for a number of different radio-tracer molecules. We furthermore show how preprocessing images with this filter improves parametric images created from such dynamic sequence.

We use zero-mean unit variance normalization, prior to performing a Hotelling filter on the slices of a dynamic time-series. The Scree-plot technique was used to determine which principal components to be rejected in the filter process. This filter was applied to [^11^C]-acetate on heart and head-neck tumors, [^18^F]-FDG on liver tumors and brain, and [^11^C]-Raclopride on brain. Simulations of blood and tissue regions with noise properties matched to real PET data, was used to analyze how quantitation and resolution is affected by the Hotelling filter. Summing varying parts of a 90-frame [^18^F]-FDG brain scan, we created 9-frame dynamic scans with image statistics comparable to 20 MBq, 60 MBq and 200 MBq injected activity. Hotelling filter performed on slices (2D) and on volumes (3D) were compared.

**Results:**

The 2D Hotelling filter reduces noise in the tissue uptake drastically, so that it becomes simple to manually pick out regions-of-interest from noisy data. 2D Hotelling filter introduces less bias than 3D Hotelling filter in focal Raclopride uptake. Simulations show that the Hotelling filter is sensitive to typical blood peak in PET prior to tissue uptake have commenced, introducing a negative bias in early tissue uptake. Quantitation on real dynamic data is reliable. Two examples clearly show that pre-filtering the dynamic sequence with the Hotelling filter prior to Patlak-slope calculations gives clearly improved parametric image quality. We also show that a dramatic dose reduction can be achieved for Patlak slope images without changing image quality or quantitation.

**Conclusions:**

The 2D Hotelling-filtering of dynamic PET data is a computer-efficient method that gives visually improved differentiation of different tissues, which we have observed improve manual or automated region-of-interest delineation of dynamic data. Parametric Patlak images on Hotelling-filtered data display improved clarity, compared to non-filtered Patlak slope images without measurable loss of quantitation, and allow a dramatic decrease in patient injected dose.

## Background

Positron emission tomography (PET) is a method to sample the distribution of an injected substance in tracer amounts and labeled with a positron-emitting isotope. PET has been used for decades as a research tool for the non-invasive assessment of biochemistry using a multitude of different tracer molecules. Recent years has seen an exponential growth of PET scanners in clinical use, mainly leveraging the radiopharmaceutical [^18^F]-Fluorodeoxyglucose (FDG) whereby a static image of a large part of the body is obtained [1].

PET can also be used as a dynamic technique, which is used to follow the distribution of a radioactive substance as a function of time, often starting at the time of injection. In dynamic PET, multiple images are acquired at different points in time, revealing information about the kinetic behavior of a substance in the body. The time-course probes the function and transport properties at the molecular level, which can be used to model different pharmacokinetic parameters, for instance using compartmental models [2]. In some research settings, continuous arterial blood sampling together with blood samples analyzed for the amount of un-metabolized (still functional) molecules are used to derive an input function to be used in the compartmental model. Simplified data-driven models exist where blood sampling is replaced by a measured uptake in a reference region directly from the images. Such models often give a good insight into the gross behavior of a molecule, where a popular method for molecules like FDG that bind irreversibly, is the reference Patlak plot [3]. The slope of the Patlak plot gives an estimate of the rate constant for specific binding, for an interaction that can be viewed as irreversible over the measurement time.

PET scanning using dynamic imaging and the various kinetic approaches are time-consuming and, when possible, the least complex methodology is sought before introduction of a new PET tracer into clinical practice. Nonetheless, the advanced methods are needed for detailed quantification of biological processes and are essential during the validation phase of new PET tracers in clinical research. For some clinical applications accurate diagnostic data requires dynamic PET scanning and kinetic modeling, for example for the evaluation of regional myocardial blood flow and oxygen consumption using [^11^C]-acetate as a tracer.

A common problem with dynamic PET techniques is that images are burdened with a high noise level, due to short measuring times of images near the injection time, and due to the decay of the radioactive isotope at later time points. When modeling is employed on each individual pixel, the described activity curve might therefore inaccurately represent the true curve. Some model parameters are highly sensitive to noise, meaning that an image with parameter values calculated for each pixel may become useless due to the amplification of noise through the model.

In principle, there are two ways to increase the signal to noise ratio in PET. One is to increase the injected dose of radioactivity to the subject. The other is to apply a filtering process to the acquired data. Increasing the radioactivity dose to a patient is always ethically dubious, especially when there is no obvious benefit to the individual as in clinical research. A standard upper limit for dose exposure to volunteers in clinical research is 10 milliSievert for estimated effective whole-body dose. With the commonly used isotope ^18^F the limit is reached after 1–2 scans, inflicting on the possibility of using PET for repeated measurements.

A number of methods have been suggested to filter out noise [4-8]. We have chosen to focus on principal component based methods, and compare our own slice-based method to volume based approaches such as in reference [8].

Principal component transforms (PCT) can be used to condense the variance in an image sequence into principal component images (or eigenimages). For PET data, principal component transforms has been shown to elucidate regional tissue features [9-11], but at the same time loosing any quantitative information.

In the imaging field, there is confusion in the use of the term Principal Component Analysis (PCA). Sometimes it means data transformed from the original dynamic image domain to the Principal Component domain (this we will call Principal Component Transform, PCT). Sometimes PCA refers to the actual filtering of data — that is, 1) applying a PCT, 2) setting some principal components to zero, followed by 3) a reverse PCT (this technique we will refer to as a Hotelling-filter). This works presents results using the Hotelling-filter as a temporal filter.

In medical imaging Hotelling-filters have been used in computed tomography and magnetic resonance imaging [12]. In dynamic PET, similar noise-reduction techniques have been described working on raw data, not images, prior to tomographic reconstruction [13]. The Hotelling-filter has been employed in PET on whole 3D volumes for the purpose of removing bias in parametric images [4], and as a temporal filter. To the best of our knowledge, the technique presented here which we have optimized for maximum noise reduction by using a single image-slice (in contrast to the whole volume) has not been applied to PET data by other groups. Parts of the current work have been presented at a conference [14].

In this paper we describe a method filtering dynamic PET data with a noise-reducing algorithm that uses the removal of principal components that have no or little discernable information. The principle of this filter is to first normalize the data to give all images equal weight, and employ the principal-component transform [15,16] on the dynamic PET image data. The second step is to remove the principal components that consist of noise, inverse the transform using the remaining components, and to reverse the normalization. What is unique with our approach is that we have chosen to perform the Hotelling-filtering on the 2D slices.

The described technique makes image sequences more visually appealing by reducing noise without degrading image resolution; it preserves quantitative information, and allows creation of certain parametric images that are otherwise too noisy to be useful. The 2D Hotelling filter is both faster and gives more accurate quantitation compared to a principal component based filter acting on the whole 3D volume.

## Methods

### Image acquisition and reconstruction

The time course of a radioactive concentration in a volume was sampled at multiple time points, using a Siemens ECAT HR+ (PET-only) scanner [17], a General Electric (GE) Discovery ST (PET/CT) scanner [18], or a GE Discovery 690 (PET/CT) scanner [19]. The described methods have been employed in a total of about 100 scans, with a large number of different tracers and diseases. A subset of these has been analyzed for this paper. The acquisition data for the cases reported in this paper will be described below.

Acquisition 1: Image data from one patient with a known cancer in the head-neck area was used. This study was part of a larger project in which we sought to evaluate the effect of treatment on cancer metabolism using serial [^11^C]-acetate PET scans. As part of this project each PET study consisted of a short dynamic scan over the heart region, immediately followed by a longer dynamic scan over the area of cancerous growth. The heart scan was used to establish an image-derived input function for subsequent kinetic modeling of acetate uptake in the tumors. Cardiac images were acquired on the GE Discovery ST [18], using 50 MBq [^11^C]-Acetate, which is only 5% of the standard dose for [^11^C]-acetate cardiac scanning in our institution. Acquisition comprised a 23 frame sequence (12×5s, 6×10s, 4×30s, 1×60s) started at injection. Images were reconstructed to a 30 cm display field of view employing standard Filtered Back Projection (FBP), with a 6.3 mm Hanning post filter.

Acquisition 2: Following the heart scan, head-and-neck images were acquired on the GE Discovery ST [18], using 1000 MBq [^11^C]-Acetate, with a 32 minute frame sequence (12×5s, 6×10s, 4×30s, 4×60, 2×120, 4×300) starting at injection. Images were reconstructed to 30 cm display field of view, using the above FBP algorithm. The same data was also reconstructed to 30 cm display field of view, using an ordered subsets-expectation maximization (OSEM) algorithm (21 subsets, 2 iterations, 3.91 mm FWHM loop filter, and 5.45 mm post-filter).

Acquisition 3: The brain images (used as one of the tracers comparing 2D and 3D Hotelling filter) were acquired on healthy volunteers, on the GE Discovery 690. A total of 250 MBq [^11^C]-Raclopride [20] was injected as bolus+infusion [21]. The 21 frame scan (9 frames × 2 min , 3 frames × 3 min, 3 frames × 4.20 min, 3 frames × 5 min) was acquired from start of injection, employing the GE Discovery 690. Images were reconstructed to a 30 cm display field of view employing the iterative VuePoint HD algorithm, with 2 iterations 24 subsets and 6.4 mm post-filter.

Acquisition 4: The brain images (used for Patlak parametric images) were acquired on the Siemens ECAT HR+ scanner [17], using 200 MBq FDG. A 90-frame scan (90 × 30s) was acquired starting at injection. Images were reconstructed to a 30 cm display field of view employing FBP, with a 4 mm Hanning post filter. The 90 images were grouped sequentially in 9 groups each containing 10 images. New 9-frame dynamic sequences were created from these groups by:

Selecting one image from each group, creating a 9-frame dynamic scan with statistics mimicking 10% (20 MBq) of the injected activity.

Summing three images from each group, creating a 9-frame dynamic scan with statistics mimicking 30% (60 MBq) of the normally injected activity

Summing all images in each group, creating a 9-frame dynamic scan from the 90-frame scan with statistics equal to 100% (200 MBq), that is, the complete injected activity.

The plasma input function was measured with arterial blood sampling.

Acquisition 5: The liver images (used for Patlak parametric images) were acquired on the GE Discovery ST using 370 MBq [^18^F]-FDG in a patient with cholangiocarcinoma and liver metastases. The scan included 16 frames (5× 60s, 5×180s, 6×300s ), acquired during 50 minutes employing 2D mode on a General Electric Discovery ST (PET/CT) scanner [18]. Images were reconstructed to a 50 cm display field of view employing FBP, with a 4 mm Hanning post filter. The plasma input function was measured with arterial blood sampling. Also an alternative image derived input function was calculated using a manually delineated region-of-interest in multiple slices of the ascending aorta, which was visible in the first frames, and clearly visible when applying the Hotelling filter.

Acquisition 6: Dynamic imaging of about 200 MBq [^18^F]-fluorothymidine (FLT) was used for determination of typical image noise levels, employing a GE Discovery 690 [19] in time-of-flight mode.

The image material was obtained from patient studies in research projects that were conducted in accordance with the declaration of Helsinki and approved by the local human ethics committee and the hospital radiation ethics committe (Uppsala-Örebro EPN reference 2006/1309-32, Uppsala University Hospital reference SEK 2005:03, and Umeå University Hospital reference 2011-263-31M). All subjects provided written consent prior to the study.

### Image processing

The new filter uses the PCA transform [16], also known as the Hotelling transform. The Hotelling filter can be applied to either the data set containing all time frames, or a subset where early frames are excluded from the principal component transforms, as described below. In the case when a subset is filtered, the final resulting data set is assembled by concatenating the original excluded time-frames with the Hotelling filtered time-frames. Thus, the resulting filtered data set will also in this circumstance contain the same number of frames as the original data set. We will now give a detailed description of the Hotelling filtering algorithm:

1) From image to principal component domain:

We organize the dynamic data for a single tomographic slice (2D Hotelling) or a volumetric matrix (3D Hotelling) into a 2-dimensional matrix, with the row vectors being pixel values for each of the *N* time-frames. We exclude the pixels that have zeros in all frames (columns with data points being all zero). Inspired by [16], each row (image) in this matrix is standardized by subtracting its mean and dividing with its sample standard deviation, giving the matrix **X**. A new matrix **A** is formed by sorting the eigenvectors of the covariance matrix of **X**, in descending order according to the corresponding normalized eigenvalues *λ*_*k*_ (one per eigenvector). The PCA transform [16] gives weighted sums **Y** of the original dynamic data, called “principal components”:

Y=AX

As an option, at this point “principal component” images can be created. This is done by putting back the excluded columns (pixels that had zeros in all frames) in their correct position in the Y matrix, and reorganizing the rows to two-dimensional images.

2) Remove noise:

The eigenvalues describe the variance of the data which is explained by the principal-component images (stored as rows in) **Y**. The eigenvectors with low eigenvalues are removed from **A**, setting the corresponding vector values to zero, giving the matrix **B**.

Thus, the first principal components 1 to *n* are used, and the components *n*+1 to *N* are set to zero. The Hotelling filter will be described as PC1-n (for instance PC1-4 if the first *n*=4 components are used).

3) From principal component domain to image domain:

The PCA transform is reversed to give an approximation **X**’ of the original images

$$X’=B^{T}Y$$

using that **B**^-1^=**B**^T^ because **B** is a matrix of orthonormal eigenvectors [15].

The standardization of the pixel values are inversed, by multiplying the rows in **X**’ by the previously calculated values of the sample standard deviation and adding the sample mean. The pixels excluded from the data vector in step 1 above (pixels that were zero in all frames) are put back in their original position. Finally, the pixels of each frame are reorganized to form a set of filtered two-dimensional images (2D Hotelling) or three-dimensional (3D Hotelling) images, where the number of filtered images is the same as the number of original time-frames.

The eigenvectors and eigenvalues were calculated using the “eig” function in Matlab, and the vectors were sorted according to their corresponding eigenvalues.

For a 27-frame, 128*128 matrix dynamic scan, producing a Hotelling filtered image takes 0.08 seconds, on a 2.6 GHz Pentium Dual-core E5300 processor.

An explanation factor *e*_*k*_ that describes the percent of the original variance, accounted for in the *k*:th principal component, is calculated

$$e_{k}=100\frac{\lambda_{k}}{\sum_{q=1}^{N}\lambda_{q}}\left[\%\right]$$

The “explained variance” is defined as the sum of the explanation factors over the *n* used principal components.

explained variance= $\sum_{k=1}^{n}e_{k}$

The explained variance is thus a measure of how much of the original variance in the dynamic image sequence remains after filtration. The suggested interpretation is that filtering two image sequences using the same number of principal components, the explained variance will be higher for the image sequence containing less noise. For a dynamic image-sequence, the explained variance increases when increasing the number of principal components employed in the Hotelling filter. The explained variance for the original image (employing all components) is 100%.

The explanation factor and inspection of the principal component images was used to select the correct number of principal components. The number of principal components to include in the Hotelling filter was determined using a Scree plot [22], that is plotting the explanation factor (or normalized eigenvalue) as a function of highest principal component (PC) number employed in the Hotelling filter. The explanation factor decreases with increasing PC number, and abruptly converges to a level close to zero. We have found that taking the PC number where the explanation factor converges to zero, and adding one gives good values for the Hotelling filter.

We have also created a second type of Scree plots, by plotting a “ROI explanation factor”, using the pixel values in the principal component images. Thus for each principal component image and ROI, a ROI explanation factor was formed as the average of the pixel values within a ROI. A Scree plot was formed by plotting these ROI explanation factors as a function of component number.

As a quality control, the residual image, defined as the difference between original image and filtered image, was inspected.

Region-of interests (ROI) were analyzed in the residual images creating plots that describe the removed uptake in a ROI as a function of time. The purpose of that kind of analysis is to find bias as a function of time. The number of principal components employed in the comparison between the 2D and 3D Hotelling filters were PC1-4.

The filtering software and Patlak slopes software was implemented as part of an in-house developed pixel-based 4-dimensional VOI tool, called imlook4d 2.00 [23], written in MATLAB 7.0 (The Mathworks, Inc., Natick, MA). The Hotelling filter is implemented as an integral part of imlook4d, giving the operator interactive feedback to any change in filtering parameters. Similarly, the principal component images and the residual (that is, the removed noise) can be viewed interactively. The parametric images were created with imlook4d, also here recalculated “on-the-fly”, to get an interactive feel when varying the 2D Hotelling filter. The 3D Hotelling filter was applied using a script to calculate a new filtered matrix.

Patlak-slope images were calculated using data starting at 20 minutes past injection. It was checked that data past 20 minutes gave a linear Patlak plot.

The displayed images and small image inserts are always displayed with equal color scales, if not explicitly stated otherwise.

### Simulations

A simulation study was performed to study dynamic data that follows a relatively large number of rate constants, a scenario we could not create with a simple radioactive phantom. We have chosen to simulate the uptake by describing typical blood and tissue uptake by mathematical functions describing the blood peak shape, and an uptake curve-shape similar to a capacitor charging curve. The reason that we did not create these by compartment modeling is that we did not have access to a true blood input functions for different tracers with varying uptake rates. Although this method can’t be claimed to be an exact representation of a specific tracer, we believe that the method allows us to freely vary time constants and thus in a simple way present more complex data than is typically present in a PET scan. Furthermore, we chose not to mimic any realistic geometry, since we did not want the reader to believe that the simulation is specific to any particular tracer uptake. Also, there is nothing in the mathematics behind the Hotelling filter that correlates with the pixel location, so we prefer to make square regions. Additional file 1 displays an example of the simulated data, and the generated activity curves.

The procedure to create this data with kinetic and noise properties similar to a dynamic PET scan will now be described. A dynamic image sequence was created consisting of 20 noise-less images, each 90 by 90 pixels. In these images, analogous to time-frames in a real PET scan, nine square areas were created, each 30 times 30 pixels in size. Each area was assigned a kinetic behavior similar to blood or tissue uptake. The tissue uptake was simulated to give different kinetic rise-times and uptake amplitudes, using the formula

$$g\left(A,k,i\right)=\mathrm{qA}\left(1-e^{-0.2\mathrm{ki}}\right)$$

with permutations of the values *A=*1,2,3, and *k=*1,2,3, where *q* is a factor that was calculated as described below. The image number *i* is an integer between 0 and 19, which represents time.

The blood uptake was calculated using the formula

$$f\left(A,k,i\right)=q\left(0.3+\mathrm{BA}e^{-\mathrm{ki}}\right)$$

The number of blood areas differed in different simulations, since we wanted to study the impact of the blood signal. We employed *k=*1 for the first blood area, *k=*2 for a second blood area, and *k=*3 for a third blood area, when applicable. The factor *B* was calculated as described below. The number of blood areas was varied between zero and three, replacing tissue areas in the image as necessary to get nine areas in the image.

Gaussian-distributed noise with a standard deviation $\sigma ={\sqrt{X}}_{i,j}$ was added to the pixel values, with *X*_*i,j*_ being the noise-less pixel values in image *i* and square area *j*. The noise was created using the Matlab “randn” function.

The factors *q* and *B* that gave the most PET-like images were determined as follows. Region-of-interests (ROIs) were drawn over tissue, lung, and blood in two FLT and two FDG dynamic scans. The mean pixel value ${\bar{x}}_{i,j}$ and the standard deviation *s*_*i,j*_ was calculated for each time-frame image *i* and ROI *j*. The relative standard deviation $s_{i,j}/{\bar{x}}_{i,j}$ was found to consistently be in the range between (0.18±0.05) to (0.58±0.10) for all four scans, calculated for each individual time-frame and ROI. The factors *q* and *B* were optimized in integer steps so that the simulated dynamic sequence matched this range of relative standard deviations. The values *q=*10 and *B=*5 gave relative standard deviations between 0.17 and 0.64 calculated for each individual test image area, and image.

Analyzing the consequences of Hotelling filtering on simulated data was done by applying a circular ROI consisting of 305 pixels within each simulated square area. The reason for using circular ROIs was only practical, since that is the default ROI-shape in imlook4d. It is plausible that filtering introduces a bias varying between early and late images, and between ROIs representing different kinetic behavior. Therefore a measure was introduced to describe the time-varying difference between noisy data (filtered or not filtered) with that of the noise-less data. This measure should have the properties that a negative and a positive difference can not cancel, so we calculated the absolute value of the noisy ROI value *x*_*i,j*_ and noise-less *X*_*i,j*_ ROI value for each ROI *j* and frame *i*. In order to get one value that summarized the bias for all different kinetics and summed over all frames we introduced

$$\mathrm{summed}\;\mathrm{bias}=100\cdot \frac{\sum_{i,j}\left|x_{i,j}-X_{i,j}\right|}{\sum_{i,j}\left|X_{i,j}\right|}.\left[\%\right]$$

with *N*=180 (20 images times 9 ROIs).

The amount of noise in Hotelling -filtered data was quantified as the standard deviation *s*_*i,j*_ of pixel values within ROI *j* from image *i*. The average standard deviation for all images and ROIs was calculated as

$$\bar{s}=\sum_{i,j}s_{i,j}/N$$

The amount of noise in the orginal noisy data was calculated using the same formula on the original data, but is referred to as *S*. The ratios of the average standard deviations of filtered and original noisy data describe the amount of noise that remains following the filtering procedure:

$$\mathrm{rs}=100\cdot \bar{s}/S\left[\%\right]$$

The spatial resolution was quantified by measuring the full-width half maximum (FWHM) under different conditions. The simulated images (which have step function interfaces between the square areas) were given a finite resolution by convoluting the image with a Gaussian shaped 2-dimensional kernel with FWHM of 4 pixels. After applying the Gaussian filter, the step-like intensity profile along a line crossing an interface thus becomes a smoothed transition between the intensity levels of the squares. Differentiating this profile gives a Gaussian-shaped profile. The FWHM of the differentiated profile was measured manually in a graph, and compared for different combinations of Hotelling-filter settings. Average values for the resolution were calculated, with error estimates being the standard deviation of these measures.

## Results

In the results section, the Hotelling settings are frequently described as for instance PC1-4. This notation means that the principal components 1 to 4 are used in the filter, whereas the values in the principal components 5 and higher are set to zero.

### Simulations

The first section of the simulation results considers the conditions when a bias may be introduced by the 2D Hotelling filtering. This is analyzed by comparing the pixel average value in a region-of-interest (ROI) in the Hotelling data with that of the noise-less original data. We have used the “summed bias” (see Methods section), a measure which basically summarizes the difference between filtered and noise-free data over all frames and kinetic curves.

**Figure 1:**
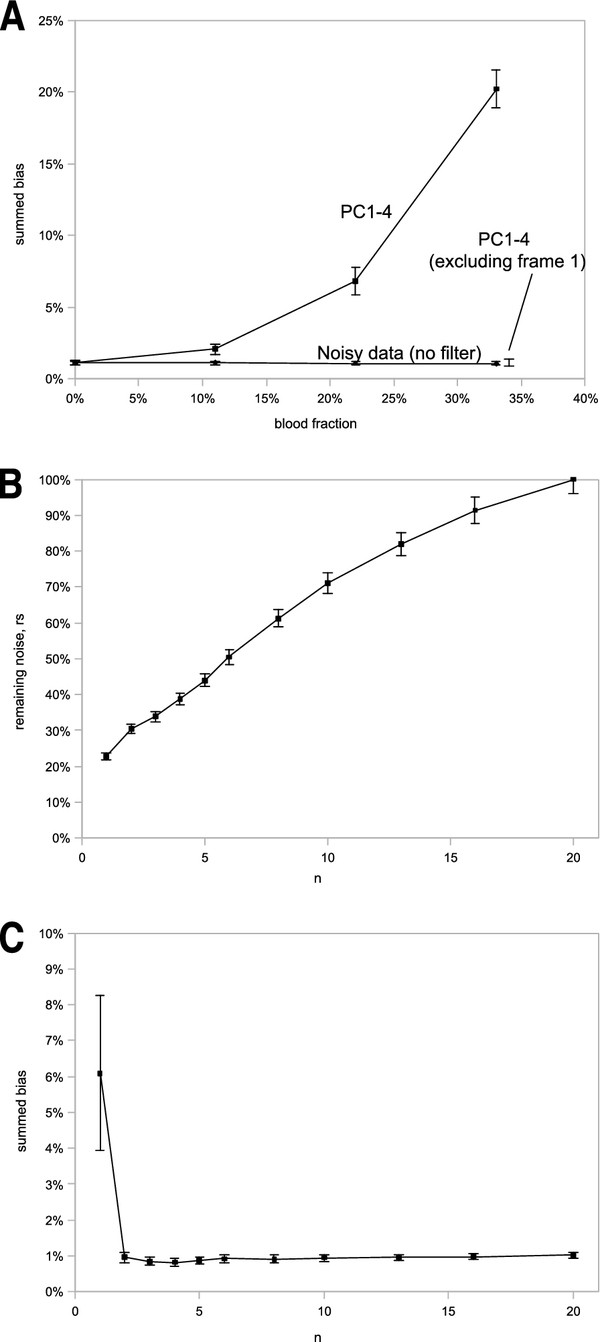
**Bias for varying percent blood pixels. A**) 2D Hotelling filtered data (PC1-4) gives increasing summed bias with increasing blood fraction (top curve). The statistical fluctuations in the summed bias, for non-filtered data, display no correlation with blood fraction (horizontal lower line). Excluding the frame with only blood activity prior to performing the Hotelling filter reduces the summed bias close to that of 0% blood (see arrow at 33%). **B**) remaining noise in ROIs of simulated data, *rs*, as a function of number *n* of employed principal components in the 2D Hotelling filter. **C**) summed bias for varying number of principal components (n=largest principal component remaining in the Hotelling filter process).

Figure 1A show the summed bias in noisy and noise-less data, with varying blood pixel fractions. The bias is constant for blood fractions between 0% and 33%.

Figure 1A also displays the bias introduced by applying Hotelling filter to a dynamic image sequence, varying the fraction of blood pixels. The bias increased with increasing fractions of blood pixels (from 0% to 33% of the pixels containing blood). Investigating individual ROIs, the absolute blood peak uptake was decreased while the early tissue uptake became falsely negative (data not shown).

Since this bias was prominent in frames where other pixels than blood showed no uptake, we made a work-around by not filtering these frames. The result was that the blood-dependent bias was removed by excluding time frames that exhibit only blood activity (first frame), before employing the Hotelling filter. Following the Hotelling filtering, the excluded image is introduced again to the beginning of the dynamic sequence. When excluding the first image from the filtering process, the resulting error was similar to that of 0% blood, and to that for unfiltered data (Figure 1A).

In summary, we have not seen any substantial bias introduced by the filtering process, when employing the Hotelling filter on data containing both tissue and blood uptake. We did however notice that a bias could be introduced when images containing only blood uptake were included.

The second section of the simulation considers the analysis of the noise removal and introduced bias, as a function of the number of principal components (PC) employed in the 2D Hotelling filter. The degree of noise removal is analyzed by comparing *rs*, a ratio of the average standard-deviations of Hotelling-filtered and original noisy data (averages performed over all dynamic images and ROIs; compare Methods section). The quantity *rs* is referred to as the “remaining noise in ROIs of simulated data”.

Figure 1B shows that only about 25% of the noise (*rs*) is left when employing only principal component 1 (PC1-1). Increasing the number of principal components employed in the Hotelling filter, *rs* increases slowly, to reach 100% when all principal components (PC1-20) are used. From the noise perspective, it seems beneficial to use as few principal components as possible, but we must also balance the selection of components and investigate biases in the ROI values as a function of number of employed PCs.

We now continue to study the results on the summed bias as a function of employed PCs. Figure 1C, shows a sudden drop in summed bias when varying the Hotelling filter from employing one component (PC1-1) to employing components 1 and 2 (PC1-2). Increasing the number of PCs further does not affect the bias in ROI values considerably. Note that the summed bias measures an average over all ROIs, and frames. This means that much larger effects may occur, as exemplified in Figure 1A. Note, that the summed bias is based on absolute values of the errors, so that we cannot expect a zero or negative value.

The third feature simulated relates to possible resolution effects. Mathematically, there is nothing that should affect spatial resolution. It is, however, possible that the limited resolution of an imaging device would smear data from different regions. It is hard to argue that this different kinetics could not affect the filtering when mixed, and therefore introduce an effective change in resolution. To simulate degraded resolution, the sequence was smeared by convolving the simulated images with a two-dimensional Gaussian kernel (sigma=4 pixels). Analyzing these smeared images according to the Methods section in this paper, a FWHM of 4.01±0.12 pixels was determined. Analyzing Hotelling-filtered (PC1-1, to PC1-4) smeared images; a FWHM of 3.87±0.09 pixels was calculated. No obvious correlation between FWHM and number of components was found.

We wish to point out that the aim of these simulations was to analyze gross effects. The exact numbers in the figures should therefore not be over interpreted.

### Comparing 2D and 3D Hotelling filter

The residual, that is the difference between filtered and original data, was investigated for each ROI. For the head-neck acetate data, 2D and 3D Hotelling filter (PC1-4) gave almost identical residuals in 4 tumor ROIs and 3 non-tumor ROIs (Additional file 2). The FDG liver data showed slightly lower residuals for 2D Hotelling filter (PC1-4) for a tumor ROI, and very similar residuals for a non-tumor ROI (data not shown).

**Figure 2:**
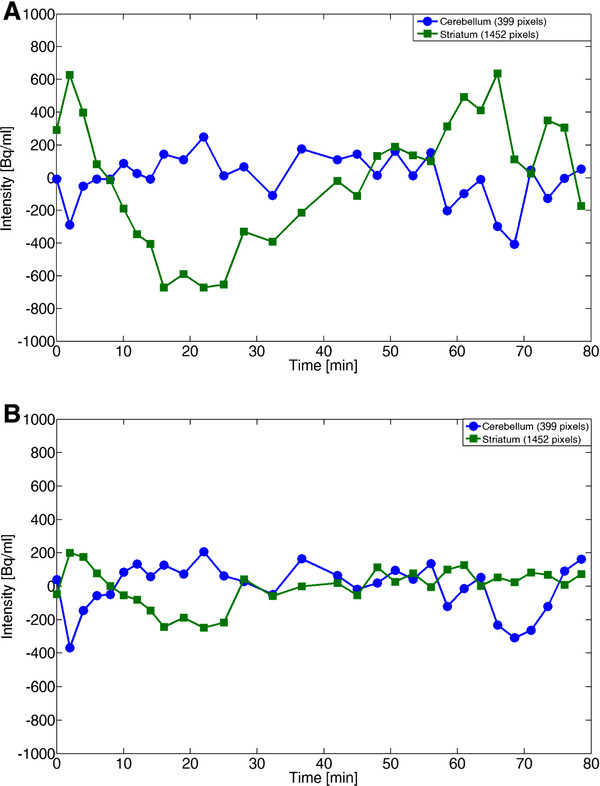
**Residuals in 2D and 3D Hotelling filter.** The residual for Raclopride plotted as a function of time for striatum and cerebellum ROIs. The residual represents the difference between filtered and original data, and is thus a measure of the bias at each time frame. **A**) 3D Hotelling filter (PC1-4), which displays a large time-varying error. **B**) 2D Hotelling filter (PC1-4), where the striatal uptake displays a considerably lower error.

The Raclopride data displays a great difference between 2D and 3D methods, where the 2D Hotelling (PC1-4) filter gives considerably less residual both in striatum ROIs and in cerebellum ROIs (Figure 2). The 3D Hotelling filter (PC1-4) displays a clear time-dependent bias for the striatum ROI (Figure 2).

### Myocardial studies using [^11^C]-acetate

**Figure 3:**
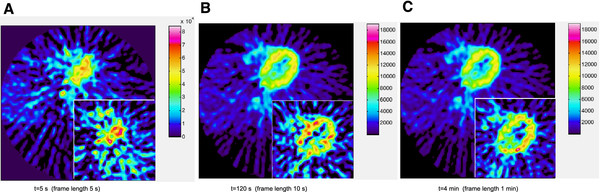
**Improved image quality, very low injected activity.** The improvement of image quality can be easily seen for a heart scan of a patient injected with only 50 MBq [^11^C]-Acetate. Large images display filtered data using principal components 1–4 (48% explained variance), and inserts show original data. The transition of acetate uptake from blood to tissue can be followed in **A**) blood in left chamber, to **B**,**C**) uptake in left chamber wall. Figure **A**) displays a 1% bias outside the circular field-of-view (corners).

The circulation of the bolus can be followed in the first few frames. Figure 3 depicts uptake in the left chamber and later uptake in the left ventricular wall. The original image is heterogeneous but becomes more homogeneous in the filtered data (employing components 1–4, 48% explained variance). The improved signal-to-noise ratio is most easily seen in later frames, but is also present especially in the background of the earlier frames.

Analyzing the region of interest for the left heart blood pool and the left ventricular wall the time-activity curves of original and filtered data agrees well (data not shown). The outlining of the region of interest could be done more accurately in the filtered data, since the bordering between regions with different uptake appeared to move around and change shape in the original images (see Figure 3B and C for comparison).

### Head and neck tumor imaging using [^11^C]-acetate

**Figure 4:**
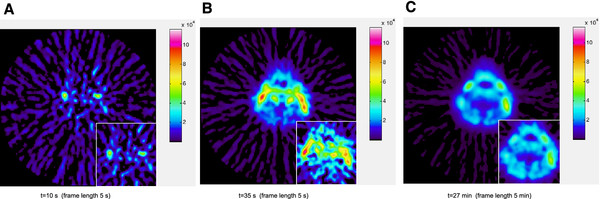
**Head neck tumour, normal injected activity.** The improvement of image quality can be easily seen for a patient examined by [11C]-Acetate. Large images display filtered data using principal components 1–4 (83% explained variance), and inserts show original data. The transition of acetate uptake from blood to tissue can be followed in **A**), only blood, **B**) blood and metastases uptake, **C**) only metastases uptake.

**Figure 5:**
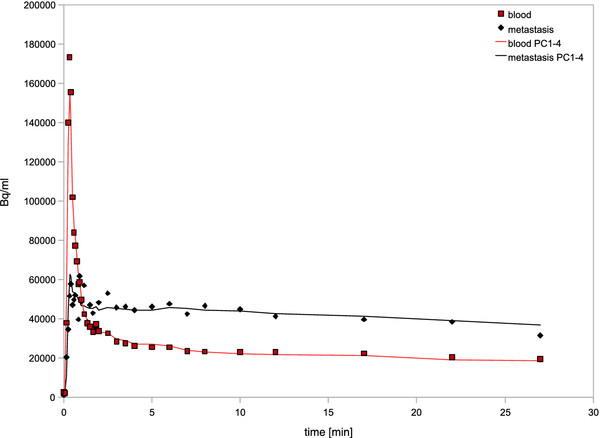
**Time-activity uptake curves.** Comparing time-activity curves in blood and metastasis region-of-interests in the same data as Figure 4. Original data (diamonds for metastases, squares for blood) and Hotelling filtered data (lines). This ROI-based time-activity graph shows preserved quantitation and the removal of noise can be seen for the metastasis curve.

The image quality is dramatically improved for all frames (Figure 4). In early frames the background noise from filtered back projection is suppressed, and in later frames the noise from statistical fluctuations due to limited radioactivity is reduced. In the filtered data, the transit of activity from the blood path to the metastases can be followed visually. Quantitation is very well preserved employing components 1-4 (89% explained variance, Figure 5), giving a time-activity curve that follows that of the original data points, but with less noise.

For both a tumor ROI and a blood ROI, the noise decreased substantially (Additional file 3). For frame 10, the blood ROI standard deviation went from original σ=1.85·10^4^ to σ=0.89·10^4^ Bq/ml (PC1-4). Also for frame 10, for a small metastasis ROI, the standard deviation went from original σ=1.59·10^4^ to σ=0.55·10^4^ Bq/ml (PC1-4).

The area under the curve is another measure that makes sense if data is going to be used for modeling. The area under the first 120 seconds of the blood curve was 148·10^6^ Bq/ml before filtering and 146·10^6^ Bq/ml following Hotelling filtering (PC1-4). The area under the metastasis curve changed from 96.8·10^6^ to 97.7·10^6^ Bq/ml for original and Hotelling filtered data, respectively.

### Parametric imaging of liver metastases using FDG

The extent of the metastases cannot easily be derived from the CT or the original PET uptake images (Additional file 4). Also, differences in Hounsfield values in the CT images do not consistently match the uptake of the PET images. For the PET images, the contrast is low because of high background uptake in normal liver.

**Figure 6:**
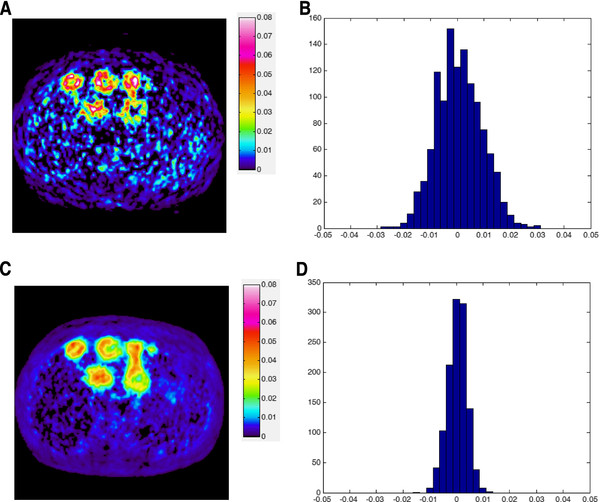
**Parametric image. A**) Patlak slope image of liver metastases using [18F]-FDG calculated employing original dynamic data. **B**) histogram of pixels in the liver for image **A**. **C**) Patlak slope image of the same data preprocessed by the Hotelling filter (PC 1–6) prior to Patlak calculations. **D**) histogram of pixels in the liver for image **C**, which displays a more narrow distribution than for the non-filtered data.

A Patlak slope image yields improved contrast between metastases and liver, but the data is noisy and the metastases are not easily delineated (Figure 6A, Additional file 4C). Applying Hotelling filter (using components 1–6) prior to the Patlak slope calculation, yields both improved contrast and reduction of noise compared to the original uptake images and non-filtered Patlak (Figure 6, Additional file 4). We speculate that the clearing of the liver uptake (Figure 6C) in the Patlak slope image may have large clinical benefit.

A histogram of the liver Patlak-slope pixel-values displays a broad distribution (standard deviation, σ=8.4·10^-3^ min^-1^) centered close to zero (0.7·10^-3^ min^-1^) (Figure 6B), whereas the distribution becomes considerably narrower (σ=3.6·10^-3^ min^-1^) but remains close to zero (0.4·10^-3^ min^-1^) when using the Hotelling filter (PC1-6) (Figure 6D). It may be noted that negative Patlak slope values does not represent irreversible binding (which is what is modeled with the Patlak analysis). The visual appearance of a bias in the Patlak-slope images that are not filtered is solely due to removing negative values (Figure 6A).

The magnitude of the Patlak slope was about 0.05 min^-1^ when applying an image derived input function making a manual ROI at the ascending aorta. When using the sampled blood plasma input function the Patlak slope values were about 2 times higher. This observation does not change anything in our analysis of the Hotelling filter, but a less naïve method than presented here should of course be applied. Examples of preferred approaches would for instance be references [24-26].

### Parametric FDG brain imaging varying injected activity

**Figure 7:**
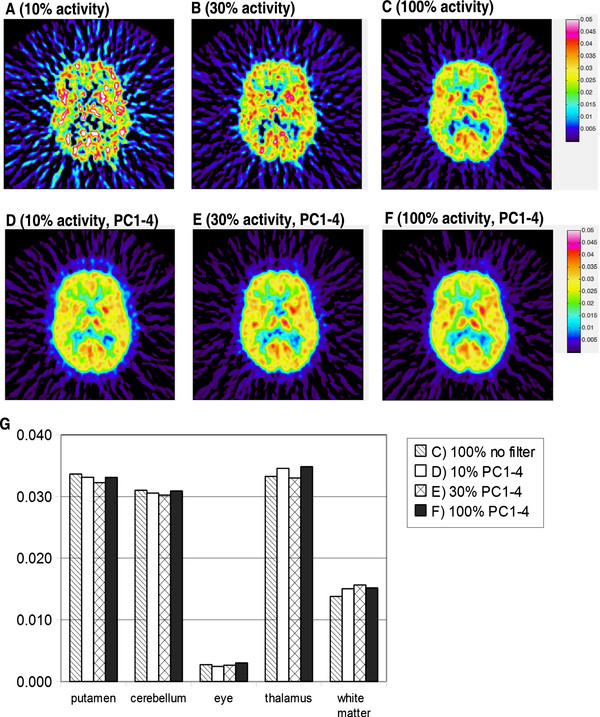
**Dose reduction. A**-**C**) Patlak slope images calculated for 10% (20 MBq), 30% (60 MBq) and 100% (200 MBq) of typical injected activity. **D**-**F**) Patlak slope images preprocessed with Hotelling filter (PC1-4) prior to Patlak slope calculations. **G**) Bar graph displaying the uptake in five regions of interests measured in images **C**-**F**.

Patlak slope images for the 10% (20 MBq), 30% (60 MBq) and 100% (200 MBq) injected activity were calculated (Figure 7A-C). The Patlak slope image for 100% activity has acceptable quality. The same data was preprocessed with Hotelling filter (PC1-4), and the Patlak slope images were calculated for 10%, 30% and 100% injected activity (Figure 7D-F). The Hotelling filtered Patlak-slope image quality is acceptable for all injected activities (Figure 7D-F), and visibly comparable to that of the non-filtered 100% data (Figure 7C). We manually delineated 5 regions-of-interest (putamen, cerebellum, the eye, thalamus and white matter). These ROI definitions were applied on the Patlak-slope images generated with Hotelling filtered data (10%, 30%, 100% activity), and 100%, non-filtered data. We found that the quantification of the Patlak slope values generated with Hotelling filter agreed within less than 10% for all injected activities to that of the 200 MBq original data.

### Quality control

**Figure 8:**
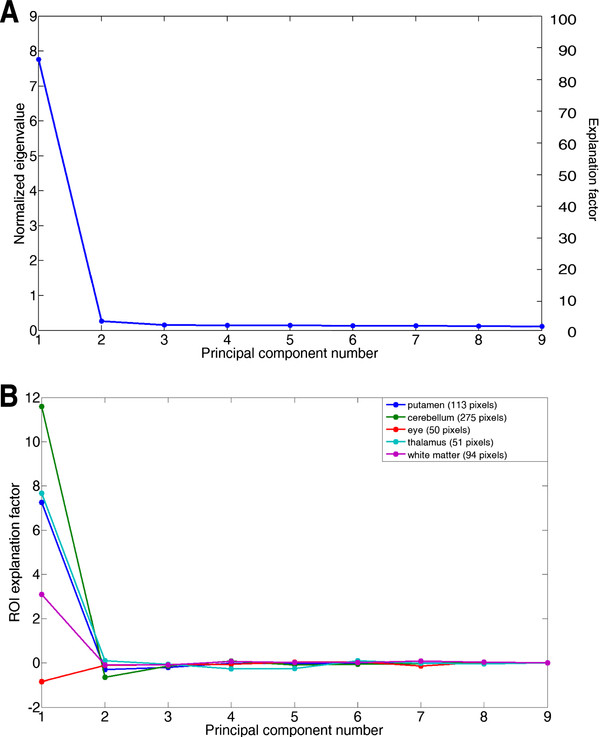
**Quality control.** Examples from the 9-frame FDG brain data (20 MBq activity): **A**) Scree plot showing the normalized eigenvalues and explanation factor as a function of the principal component number n. **B**) A ROI explanation factor plot showing the average pixel value from the principal component images, of five regions-of-interest, plotted as a function of principal component number n. The interpretation is that n equals 4 is a good selection for this data, since the intensities for all ROIs have converged to near zero at n=4.

The intensity explained in different principal components was investigated using a Scree plot [22]. The plot was constructed by plotting the explanation factor as a function of component number (Figure 8A). In all cases investigated, it was observed that the explanation factor decreases with higher principal component, and converges to a value close to zero.

After defining regions of interest, a second form of Scree plot was constructed by analyzing ROIs in the principal component images. The ROI explanation factor was plotted, for each ROI, as a function of component number (Figure 8B). This region-of-interest based Scree plot works well to pinpoint if an anatomical region requires more principal components than others. For instance, it was found in the FDG Patlak slope work that the nose uptake was significant in principal component 3, but had decreased to close to zero in component 4 (Figure 8B). This was the reasoning for filtering that data with components PC1-4.

In another form of quality control the residual image, defined as the difference between original image and filtered image, was calculated. Observing how the residual image varies with increasing number of included components, pixel values in the anatomical region goes from being highly correlated to noisier (Additional file 5). The principal component where anatomical information in the residual image stabilizes with increasing component seems to coincide with the component following where the curve in the Scree-plot bends to nearly horizontal. The conclusion is that the residual image is a possible quality control criterion. One thing to remember is that the noise typically increases with (square root of) amplitude. This will naturally cause high-uptake regions not visible in the uptake image to be accentuated in a residual-image. Thus higher-uptake areas will in the residual image show higher minimum and maximum values, than will the low-uptake regions. This is not to confuse with a badly applied filter, but is statistically unavoidable. The test for this is to look at the ROI-value in the residual image (or ROI explanation factor plot), which should approach zero when enough principal components are employed.

## Discussion

The 2D Hotelling filter enhances image quality in existing dynamic PET scans, without loosing quantitative information. This could open up various new opportunities. A potential major application, as demonstrated in this paper, could be a substantial radiation dose reduction which might reduce risk to patients and healthy volunteers. This lower dose also implies the potential to perform multiple scans on the same subject, without exceeding standard dose limits.

Alternatively, the Hotelling filter could be utilized to improve image quality with existing levels of injected radioactivity. This enhanced image quality allowed the creation of parametric images, that in some cases were clinically unreliable when created using non-filtered images. A major benefit with this method would be that it is possible to implement on any computer, can be used on images from existing scanners and would be independent of scanner manufacturer.

There does not seem to be any mathematical mechanism in the principal component analysis that affects the image resolution, and the simulation studies showed this to be true within the limits that our analysis permits. The Hotelling filter therefore does not seem to suffer from the resolution degrading of traditional low pass filters. Visually, the effect of removing noise actually gives an appearance of improved resolution, probably due to the better contrast achieved. One application of this is that filtered images can give more reproducible region-of-interest delineation.

To derive some types of parametric images, the blood input function is required. The improved image quality for low injected activity (50 MBq) heart study showed that the blood pool can be delineated. Thus, without adding any significant dose to the patient, a time activity curve of the arterial blood could be acquired prior to a second scan. Later, a second injection with higher activity can be performed allowing a second scan over another body part. Assuming that systemic circulation is unchanged, this allows for kinetic modeling without any arterial blood sampling, potentially increasing the clinical appeal of kinetic modeling techniques in PET.

Since the principal-components are sorted according to the variance, it is hard to infer if a large area with small variance or a small area with large variance will get precedence and be explained in a higher-order principal component. However, the beauty of the Hotelling filter is that the order of the components is not important as long as the significant components are included in the filter. This is secured by inspecting the Scree plot either for the whole image, or preferably for the important regions of interest (ROI explanation factor plot). A trained operator may learn to do this Scree test visually directly in the image domain, by increasing the number of components while inspecting when the features in the image becomes stable. Features in the residual image (the removed noise) can also be interactively compared with the original image while adjusting the number of principal components, and only unordered noise should remain. Worth noting is that using the residual image is a mere visual inspection, and it is not an obvious measure. Furthermore, it requires a trained operator to tell the high noise level from a high-uptake region apart from correlated uptake. Hence, we would recommend using the Scree tests, in both forms.

Observing the residual image (and also the principal component images), we have found that small structures can be lost in the Hotelling filter. The reason seems to be that these structures appear in lower order components, even though their kinetic patterns are unique and with high amplitude. This is part of the principal component algorithm, where the total variance in a region with its own kinetic is compared to the variance in the whole image. This should not be specific to any particular tracer, but to the size of a region and its uptake amplitude. We believe that this should be studied further in order to make it possible to minimize the manual inspection of ROI-based Scree plots and residual images.

We have found that a good work flow to avoid missing small structures (see above paragraph) is to use the possibilities in the imlook4d [23] software to vary the number of employed principal components using the computer mouse, while inspecting the Hotelling-filtered image. For a trained radiologist, it is easy to follow the decrease in noise while lowering the number of used principal components. At a point when features start to change in intensity, too many components are removed, and the radiologist then goes back until the features are back. This whole sequence takes a few seconds to perform. It is also possible to view the residual images (that is the data that is removed by the filter) while varying the number of used principal components.

If quantification is to be performed, drawing regions-of-interest can be done more easily on the Hotelling-filtered images. Quality control is then done creating a ROI explanation factor plot for these particular ROIs. The procedure to do this is to view the principal-component (PC) images, and performing a “time-activity plot” (which makes the Scree plot for the ROIs when the imlook4d software is in PC-image mode).

Comparing the 3D Hotelling filter to the 2D Hotelling filter, the focal uptake in Raclopride gave a large time-dependent bias in the 3D Hotelling. This may be explained by the observation in the above paragraph. The striatum is represented by a much smaller fraction of the pixels in the 3D volume than in the 2D slice, and therefore the striatum is in the 3D case represented by several lower principal components. Thus, parts of the uptake may be lost when removing components. The alternative approach, to remove fewer principal components in the 3D Hotelling filter would render less noise reduction.

From the presented results, we believe that the 2D Hotelling filter is more suitable to perform noise-reduction in a quantitative way, than is the traditionally applied 3D filter. Also, since the 2D and 3D Hotelling filter seems to perform equally for other tracers, we believe that the 2D Hotelling filter is the safest method.

In order to validate the filter for clinical use for a specific tracer and part of the body, the filtering parameter may be varied in a sufficiently large sample of patients. With this approach we have found that for the presented tracers, and 10 or more time frames, using Hotelling filter (PC1-4) preserved quantitation and reduced image noise. This process is similar to the validation of any other reconstruction parameters, for instance the optimization of the number of iterations and subsets in an iterative reconstruction.

The principal-component transform is sensitive to patient movement. We have observed that using the typical Hotelling filter (PC1-4), patient movement induces edge effects in the filtered data. These effects can also be clearly seen in the residual image. Increasing the number of employed principal components, the edges disappear but because we use more principal components the noise level in the filtered images go up and we gain less. It is therefore important to either immobilize the patient or to realign the different time-frame images to each other. Based on this observation, we speculate that principal component analysis might be possible to use as a tool to spot uncorrected motion.

The presented filter has, apart from PET, been tried on dynamic SPECT, MR and CT and the images appear much nicer. Employing the Hotelling filter in the above applications may allow injections with lower activity, or could be used to enhance image quality.

## Conclusions

The 2D Hotelling-filtering single-slice dynamic PET data is a computer-efficient method that gives improved differentiation of different tissues due to removal of noise. We have also observed that for the focal uptake in Raclopride, the 2D filter is preferred compared to the volumetric 3D filter. We have found that the Scree-plot, combined with the ROI explanation factor plot, is a simple interactive and fast method to determine the number of principal components to employ. Parametric Patlak images on Hotelling-filtered display improved clarity, compared to non-filtered Patlak slope images without loss of quantitation. We have for FDG Patlak slope images of brain, shown that a substantial patient dose reduction is possible with no or little loss of quantitation.

## Competing interests

JA and JS have both been employed by Uppsala Imanet during parts of the last 5 years (Uppsala Imanet was owned by General Electric, GE Healthcare). JA has during this employment applied for a patent which in parts claims a version of the described Hotelling filter, as part of a broader context. See U.S. Patent Application No. 12/745,739, Publication No. 2010/0260402 (published Oct. 14, 2010)(Jan Axelsson and Anna Ringheim). We do however believe that the described work is in its own right not part of this patent application because of a) prior art (Med Phys 21:193-201,1999) and b) the method described.

## Authors’ contributions

JA came forward with the initial idea, and carried out programming and analysis. JS selected medically interesting scans that we had consent to use, advised with medical understanding and performed the medical conclusions. The writing of the article was shared between JA and JS. The article would not have been conceived without the joint work of JA and JS. Both authors read and approved the final manuscript.

## Pre-publication history

The pre-publication history for this paper can be accessed here:

http://www.biomedcentral.com/1756-6649/13/1/prepub

## Supplementary Material

Additional file 1 **Simulation data.** Simulated data for frame 3, 33% blood pixels. Blood regions are regions 1, 4 and 7 in image A. **A**) Noise-less simulated data. ROI numbers are indicated in the figure. **B**) Simulated data with applied noise. **C**) Activity curves for noise-less data (A) **D**) Activity curves for noisy data without filtering. Click here for file

Additional file 2 **Residuals in 2D and 3D Hotelling filter.** Activity plotted as a function of time (logarithmic time scale) of the residual for 4 tumour ROIs for the Acetate head-neck data: **A**) 3D Hotelling filter PC1-4. **B**) 2D Hotelling filter PC1-4. Click here for file

Additional file 3 **Histograms.** Example of histograms measured in volume-of-interests from frame 10 in the head-neck acetate data: **A**) Blood pixel values in original data. **B**) Blood pixels in Hotelling filtered data (PC1-4), displaying a much more narrow distribution than in A. **C**) Metastasis pixels original. **D**) Metastasis pixels in Hotelling filtered data (PC1-4), displaying a much more narrow distribution than in C (PDF 111 kb) Click here for file

Additional file 4 **Liver metastases imaged with CT, and dynamic FDG PET. A**) CT shows intensity variations (Hounsfield units) that do not completely overlap PET uptake. A square is drawn in the CT and PET images to guide the eye. **B**) PET uptake 45 minutes post injection (5 minute frame duration), in units Bq/ml. **C**) Patlak slope image of original data, in units min^-1^. **D**) Patlak slope image of Hotelling filtered data (PC 1–6), in units min^-1^. Click here for file

Additional file 5 **Residual images.** An example of the use of residual images for quality control, applied to the cardiac study. **A-C**) display data filtered using components 1–2, 1–4, and 1–6, respectively. **D-F**) display the residual images, that is, the data that was removed in the filtering process. All intensity scales are in unit Bq/ml. It can be noted that in **D**) both positive and negative homogeneous residual areas exist, which suggest that not enough principal components are used. In **E**) the homogeneous negative residual area has been exchanged with noise, and the images do not appear to change when further increasing the number of components (**F**). The observed larger residual-fluctuation in pixels with high uptake is not surprising, since large uptakes accommodate higher noise amplitudes (even though they are visually harder to notice in the uptake images). Click here for file
