# Supplementary figures and images for: The 2D Hotelling filter - a quantitative noise-reducing principal-component filter for dynamic PET data, with applications in patient dose reduction
Source: BMC Med Phys. 2013 Apr 10;13:1. doi: 10.1186/1756-6649-13-1 (PMC3636030; doi:10.1186/1756-6649-13-1)

**A**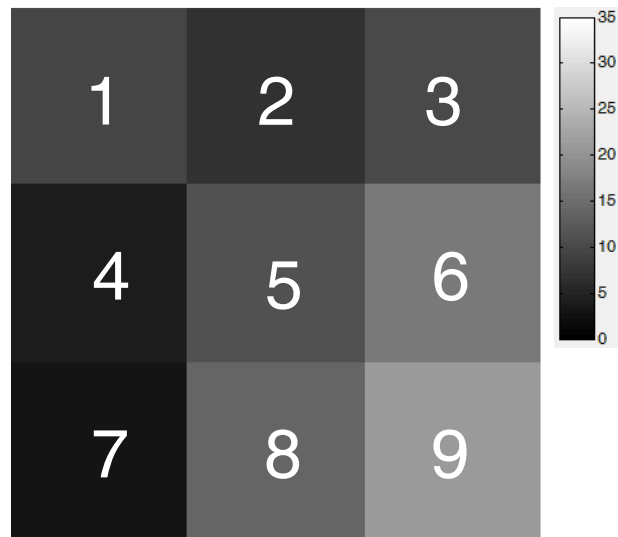**B**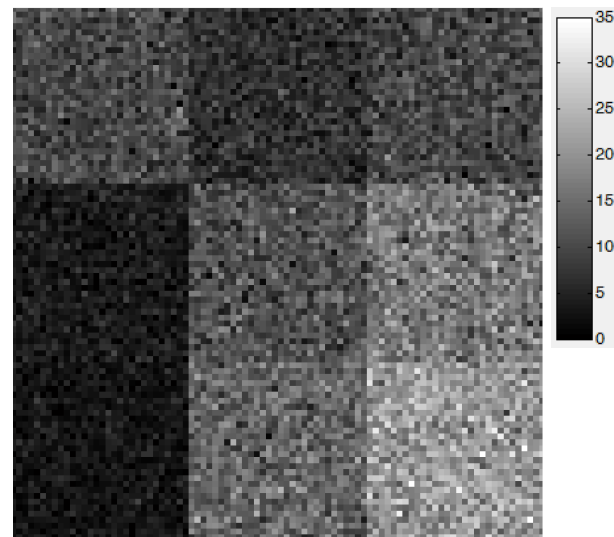**C**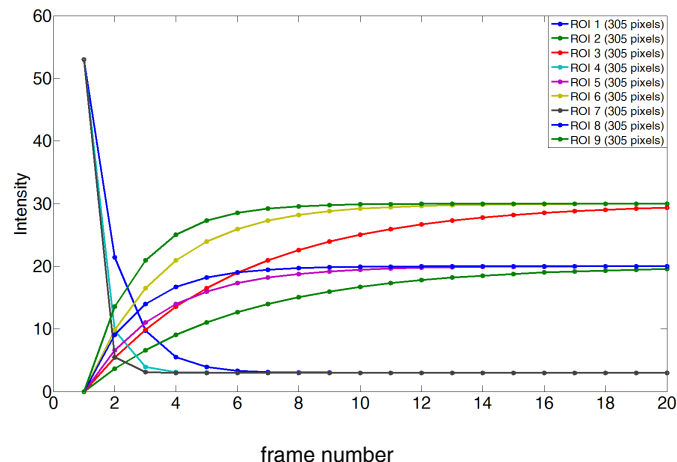**D**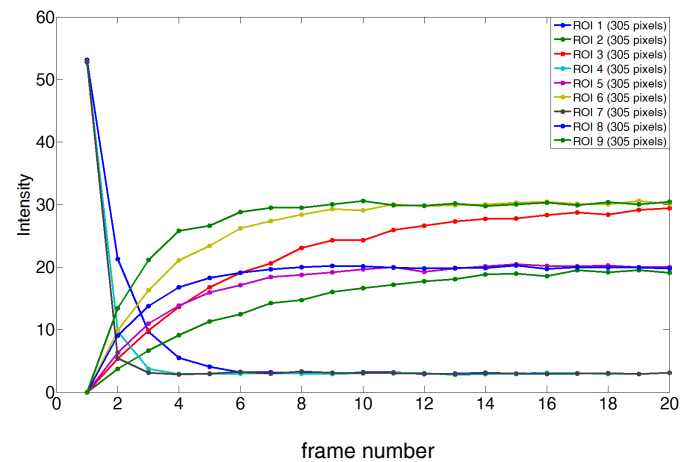

Supplement: Additional file 1 — Simulation data. Simulated data for frame 3, 33% blood pixels. Blood regions are regions 1, 4 and 7 in image A. A) Noise-less simulated data. ROI numbers are indicated in the figure. B) Simulated data with applied noise. C) Activity curves for noise-less data (A) D) Activity curves for noisy data without filtering. [file 1756-6649-13-1-S1.pdf]

**A**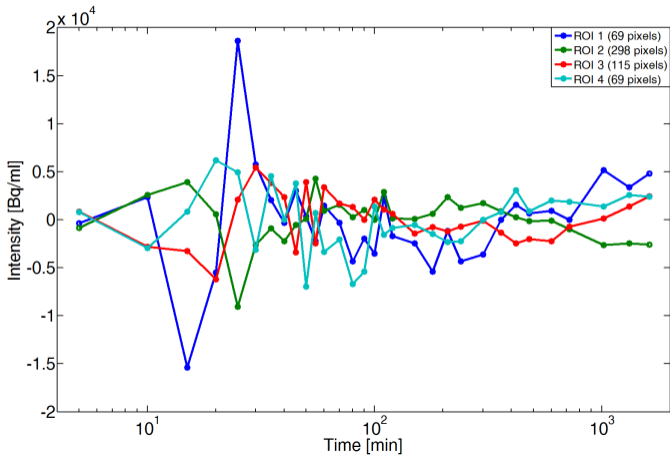**B**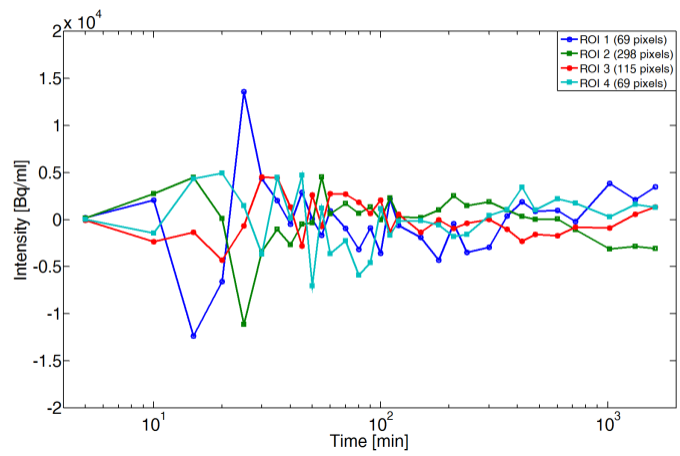

Supplement: Additional file 2 — Residuals in 2D and 3D Hotelling filter. Activity plotted as a function of time (logarithmic time scale) of the residual for 4 tumour ROIs for the Acetate head-neck data: A) 3D Hotelling filter PC1-4. B) 2D Hotelling filter PC1-4. [file 1756-6649-13-1-S2.pdf]

**A**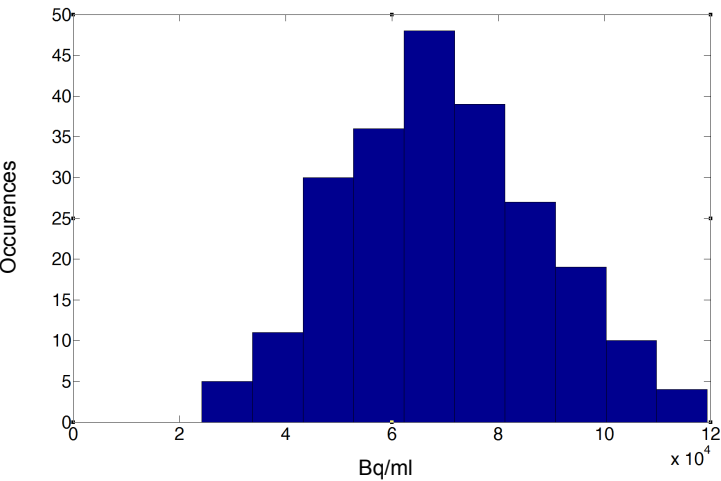**B**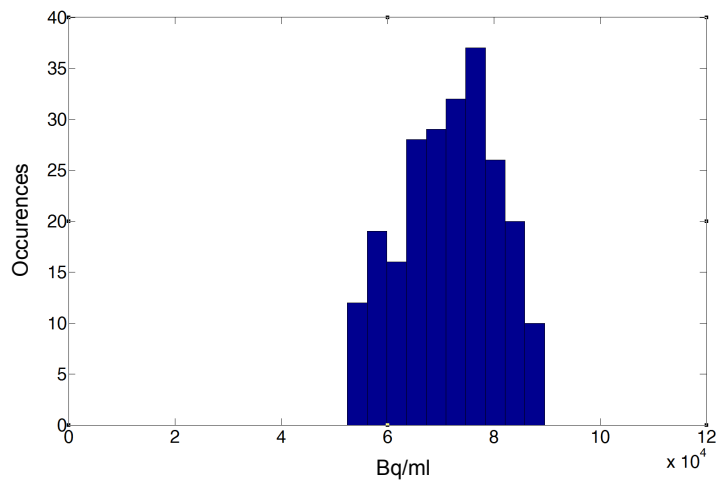**C**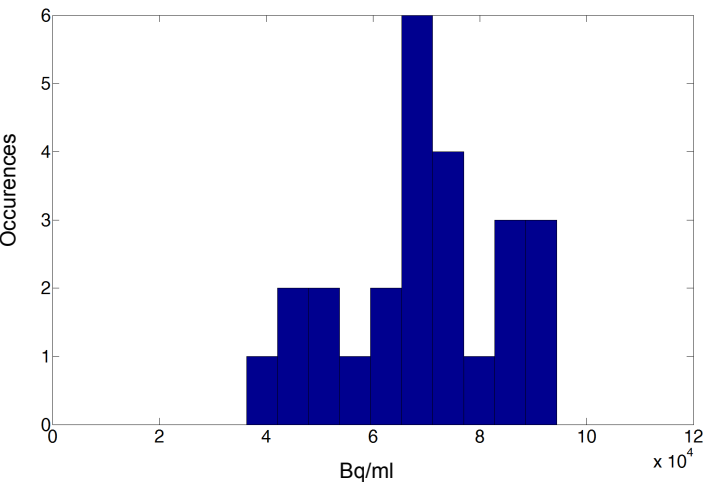**D**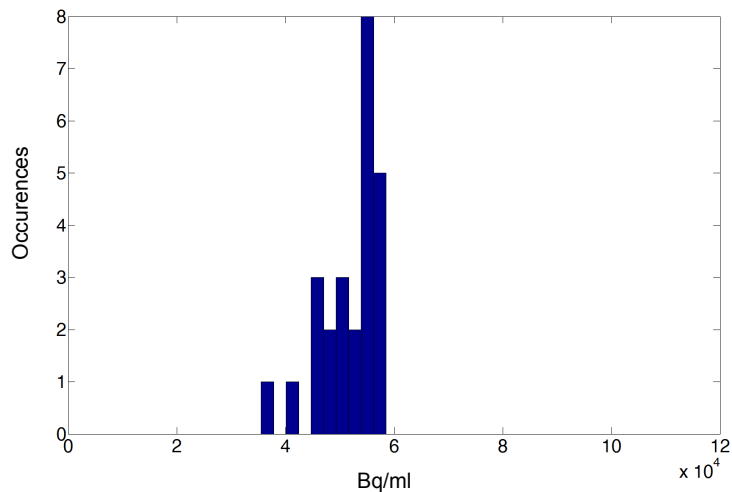

Supplement: Additional file 3 — Histograms. Example of histograms measured in volume-of-interests from frame 10 in the head-neck acetate data: A) Blood pixel values in original data. B) Blood pixels in Hotelling filtered data (PC1-4), displaying a much more narrow distribution than in A. C) Metastasis pixels original. D) Metastasis pixels in Hotelling filtered data (PC1-4), displaying a much more narrow distribution than in C (PDF 111 kb) [file 1756-6649-13-1-S3.pdf]

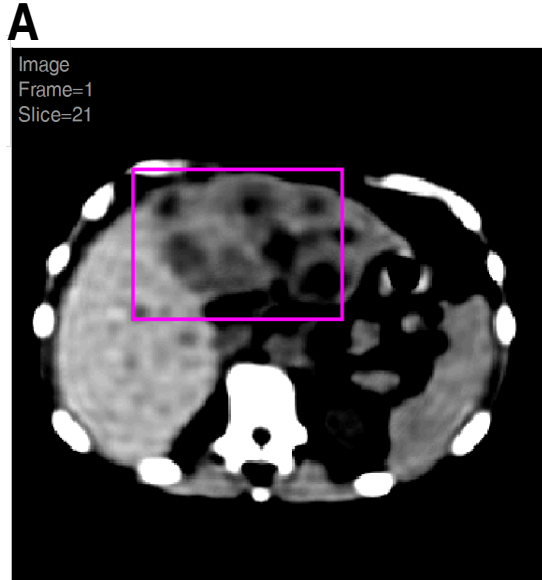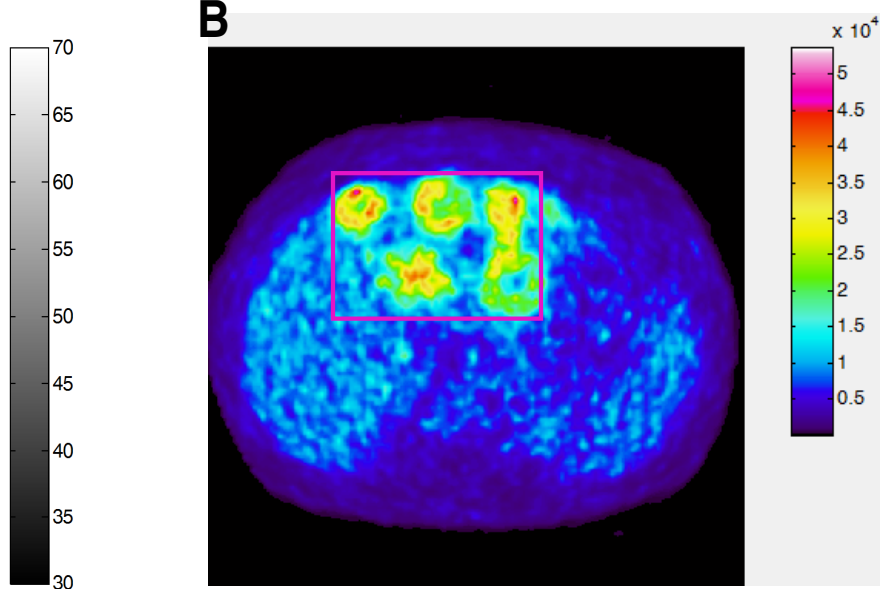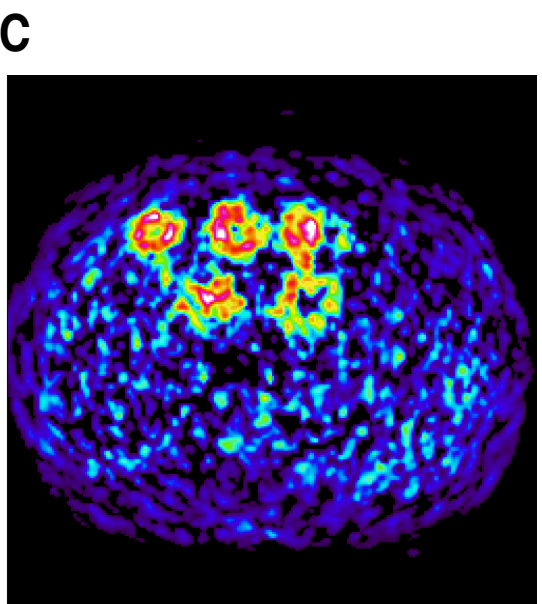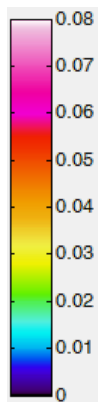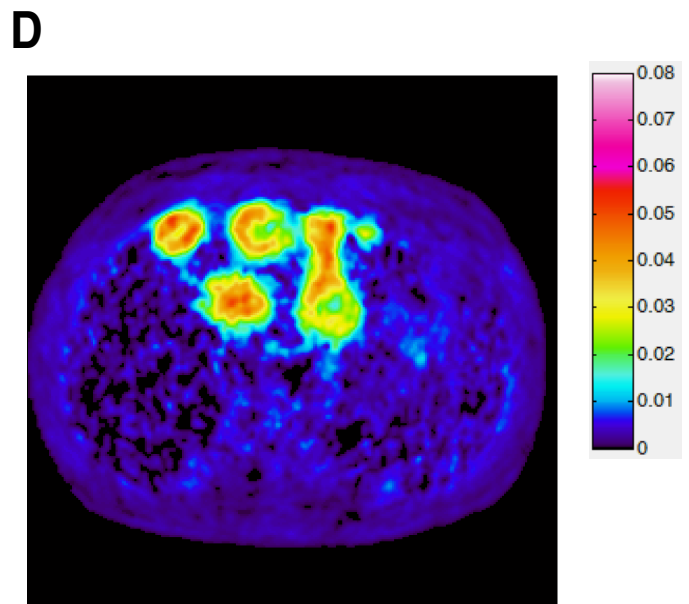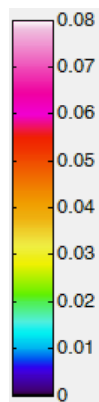

Supplement: Additional file 4 — Liver metastases imaged with CT, and dynamic FDG PET. A) CT shows intensity variations (Hounsfield units) that do not completely overlap PET uptake. A square is drawn in the CT and PET images to guide the eye. B) PET uptake 45 minutes post injection (5 minute frame duration), in units Bq/ml. C) Patlak slope image of original data, in units min-1. D) Patlak slope image of Hotelling filtered data (PC 1–6), in units min-1. [file 1756-6649-13-1-S4.pdf]

**A**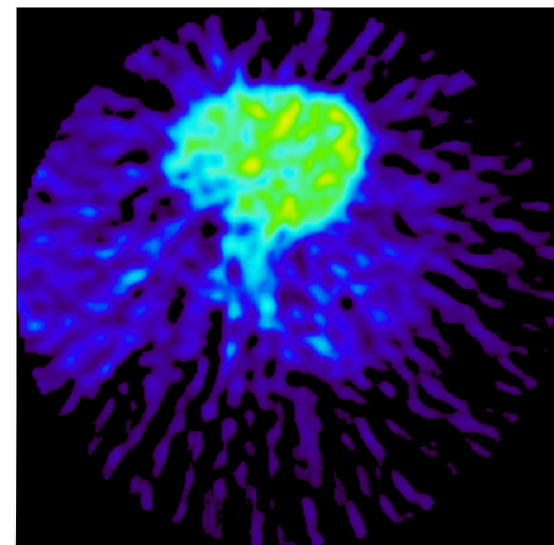

PC1-2

**B**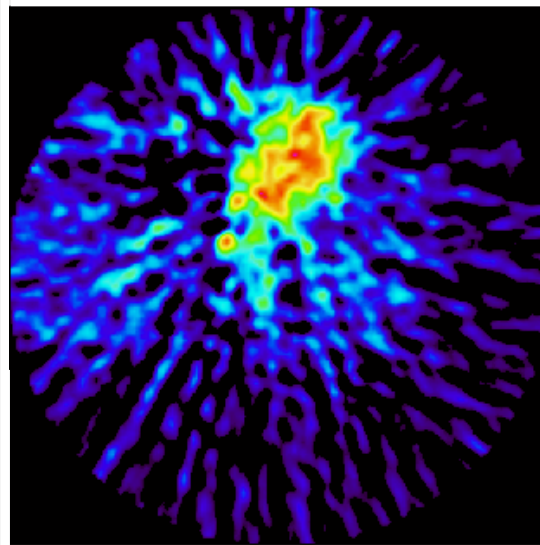

PC1-4

**C**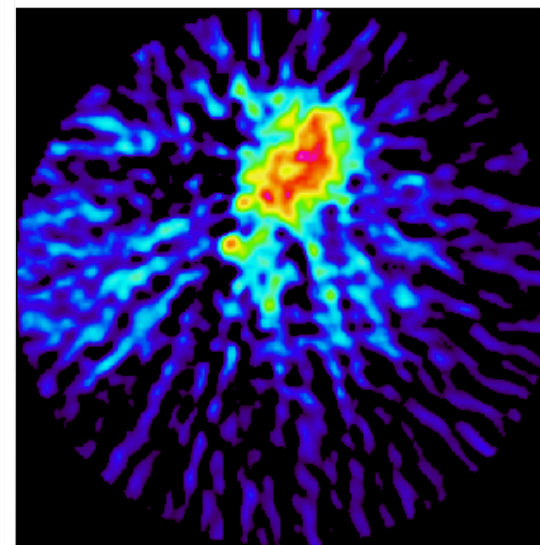

PC1-6

**D**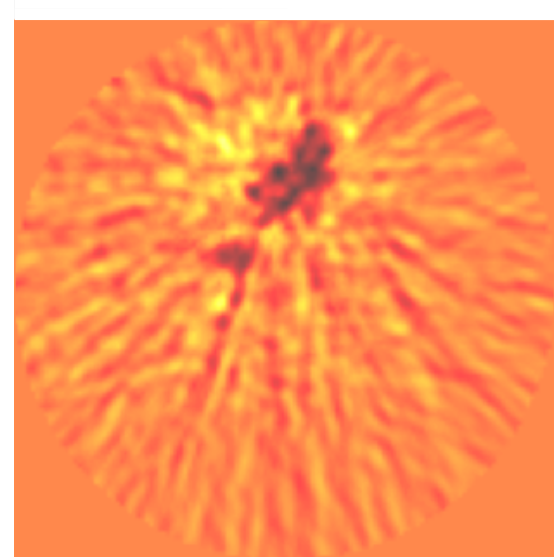

Residual PC1-2

**E**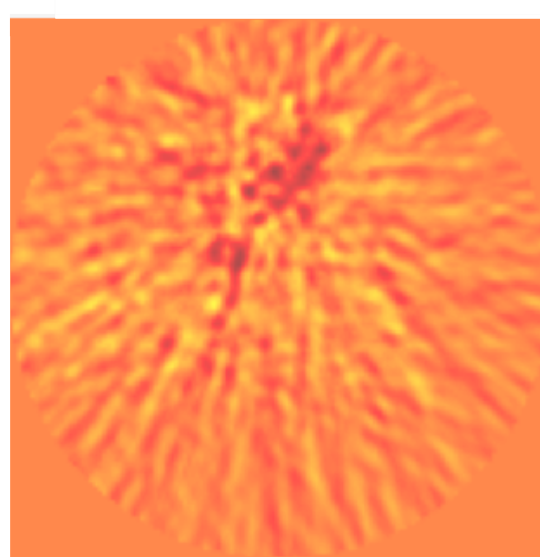

Residual PC1-4

**F**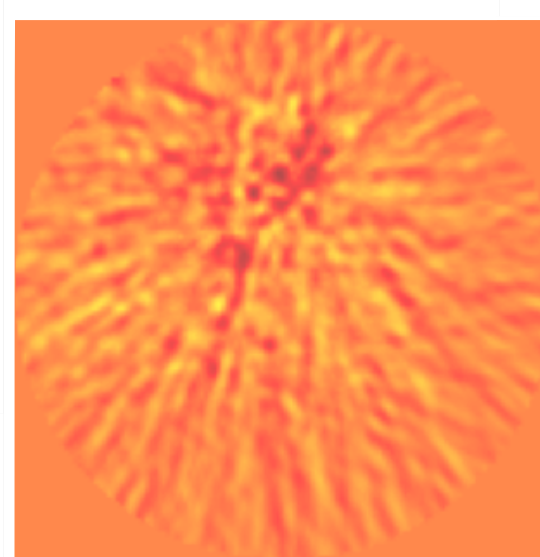

Residual PC1-6

Supplement: Additional file 5 — Residual images. An example of the use of residual images for quality control, applied to the cardiac study. A-C) display data filtered using components 1–2, 1–4, and 1–6, respectively. D-F) display the residual images, that is, the data that was removed in the filtering process. All intensity scales are in unit Bq/ml. It can be noted that in D) both positive and negative homogeneous residual areas exist, which suggest that not enough principal components are used. In E) the homogeneous negative residual area has been exchanged with noise, and the images do not appear to change when further increasing the number of components (F). The observed larger residual-fluctuation in pixels with high uptake is not surprising, since large uptakes accommodate higher noise amplitudes (even though they are visually harder to notice in the uptake images). [file 1756-6649-13-1-S5.pdf]
